# Generation of Influenza Virus from Avian Cells Infected by *Salmonella* Carrying the Viral Genome

**DOI:** 10.1371/journal.pone.0119041

**Published:** 2015-03-05

**Authors:** Xiangmin Zhang, Wei Kong, Soo-Young Wanda, Wei Xin, Praveen Alamuri, Roy Curtiss

**Affiliations:** 1 The Biodesign Institute, Arizona State University, Tempe, Arizona, United States of America; 2 Department of Pharmaceutical Sciences, Eugene Applebaum College of Pharmacy/Health Sciences, Wayne State University, Detroit, Michigan, United States of America; 3 School of Life Science, Arizona State University, Tempe, Arizona, United States of America; University of Geneva, SWITZERLAND

## Abstract

Domestic poultry serve as intermediates for transmission of influenza A virus from the wild aquatic bird reservoir to humans, resulting in influenza outbreaks in poultry and potential epidemics/pandemics among human beings. To combat emerging avian influenza virus, an inexpensive, heat-stable, and orally administered influenza vaccine would be useful to vaccinate large commercial poultry flocks and even migratory birds. Our hypothesized vaccine is a recombinant attenuated bacterial strain able to mediate production of attenuated influenza virus *in vivo* to induce protective immunity against influenza. Here we report the feasibility and technical limitations toward such an ideal vaccine based on our exploratory study. Five 8-unit plasmids carrying a chloramphenicol resistance gene or free of an antibiotic resistance marker were constructed. Influenza virus was successfully generated in avian cells transfected by each of the plasmids. The *Salmonella* carrier was engineered to allow stable maintenance and conditional release of the 8-unit plasmid into the avian cells for recovery of influenza virus. Influenza A virus up to 10^7^ 50% tissue culture infective doses (TCID_50_)/ml were recovered from 11 out of 26 co-cultures of chicken embryonic fibroblasts (CEF) and Madin-Darby canine kidney (MDCK) cells upon infection by the recombinant *Salmonella* carrying the 8-unit plasmid. Our data prove that a bacterial carrier can mediate generation of influenza virus by delivering its DNA cargoes into permissive host cells. Although we have made progress in developing this *Salmonella* influenza virus vaccine delivery system, further improvements are necessary to achieve efficient virus production, especially *in vivo*.

## Introduction

Influenza viruses belong to the family *Orthomyxoviridae* and contain single-stranded segmented RNA genomes. They are classified into types A, B, and C based on the serological differences in the nucleoproteins (NP) and matrix proteins [1]. Unlike type B and C influenza viruses which infect very limited species [2,3], influenza A virus has a broad host range including humans, avian species, and a diversity of mammals [4–6]. As of today, influenza A virus with all hemagglutinin/neuraminidase (HA/NA) combinations are found in aquatic bird reservoirs [4,7]. Without causing apparent signs of disease (except some H5N1 strains), the virus replicates in the respiratory and intestinal tracts of the aquatic birds [8,9], where the cells mainly express α2–3 linked sialic acid (SA) receptors [10]. After being excreted in high concentration in the feces, influenza A virus remains infectious in lake water for up to 4 days at 22°C and more than 30 days at 0°C [11]. Backyard poultry have chance to contact with migratory birds, occasionally become infected through the fecal-oral route and introduce new “foreign” influenza viruses [12–14]. The foreign virus therefore causes an outbreak among immunologically naïve birds. The world-wide outbreaks of highly pathogenic avian influenza (HPAI) H5N1 have resulted in death or depopulation of wild birds and poultry in hundreds of millions [15]. Additionally, the avian virus could be transmitted to humans from infected poultry at low efficiency, such as occurred for the H5N1 avian influenza virus [16–18]. This low infectivity was most likely because the avian virus prefers α2–3 linked SA receptors which only reside in the less reachable lower respiratory tract of humans [19]. After adaptation in infected humans or other mammalian hosts, the virus may acquire the ability of efficient binding to α2–6 linked SA receptors in human trachea as the human influenza virus does [20]. Another possibility is that an intermediary host serves as the mixing vessel [21]. For instance, pigs have α2–3 and α2–6 linked SA receptors in the trachea [22] and is susceptible to avian and human influenza viruses [23–25]. Dual infection with both viruses can result in reassortant viruses [26,27]. By either way, the viruses with foreign HA become human-to-human transmissible and may result in an epidemic or pandemic in the human population.

In numerous countries, inactivated influenza virus vaccines have been used to combat the highly and low pathogenic avian influenza viruses [28–33]. Such measures not only benefit the poultry and egg industry but also prevent virus transmission to humans. However, manufacturing the current veterinary influenza vaccine relies on healthy chicken embryos [33]. Administration of the vaccine by subcutaneous or intramuscular injection is costly and labor-intensive, and thus is not ideal for vaccinating large commercial flocks and wild birds, leaving slaughter of infected poultry as the viable option to control the spread of avian influenza.

**Fig 1:**
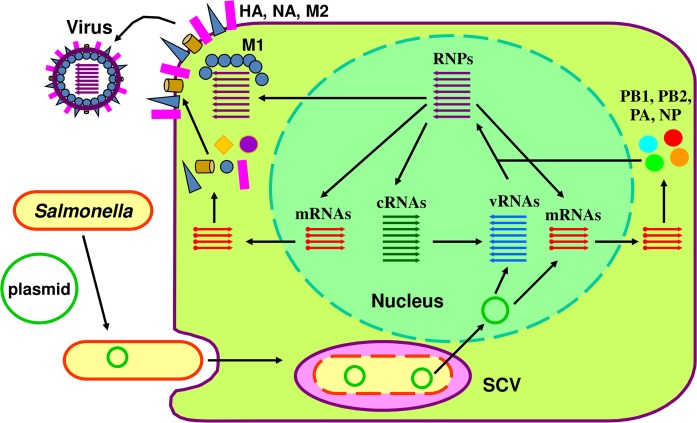
Schematic representation of recombinant *Salmonella*-mediated generation of influenza virus. A recombinant attenuated *Salmonella* strain carrying the 8-unit plasmid invades host cells and stays in the *Salmonella*-containing vacuole (SCV). Due to the lack of essential supplements (e.g. DAP or D-alanine) for cell wall synthesis, the replicating bacteria undergo cell wall-impaired lysis. The plasmid is released and translocated into the host cell nucleus, where it serves as a template for the transcription of vRNAs (whole genomic set) and mRNAs (for PB1, PB2, PA and NP). The mRNAs are translated into proteins to bind vRNAs to form the complete set of RNPs. RNPs direct the synthesis of cRNAs, which in turn serve as templates for vRNA production. RNPs also direct the synthesis of mRNAs for HA, NA, M and NS in addition to mRNAs for polymerase and the NP. The nascent HA, NA and M2 target the host cell membrane. The resulting new virus buds from the host cell and is capsulated with membrane embedded with HA, NA and M2.

Live bacterial vaccines are effective, safe, inexpensive, and can be rapidly produced in large quantities [34,35]. The other distinctive feature is immunization via the oral or intranasal routes, thus reducing the operational costs involved in administering injectable vaccines. *Salmonella* is one of the most promising bacterial vectors that can deliver target protective antigens through the host mucosa [36]. Numerous technologies have been developed over the years to make more safe and effective *Salmonella* vectors for various applications, such as programmed *Salmonella* lysis for delivery of protein or plasmid cargoes [37–39], balanced lethal systems for *Salmonella* to harbor plasmid free of antibiotic resistance genes [40–45], regulated delayed *in vivo* attenuation [46,47], and *recA* or *recF* deletion to stabilize plasmids containing repetitive sequences [48]. On the other side, we had created an 8-unit plasmid that encodes vRNAs and proteins for assembling influenza virus in avian cells [46]. In this study we integrated the *Salmonella* delivery methodology with the pure DNA based-reverse genetics of influenza virus [49,50]. Several recombinant attenuated *Salmonella* strains were generated to stably harbor and deliver the 8-unit plasmid into the host cells to recover influenza virus as illustrated in Fig. 1. Our data shed light on the development of a novel vaccine composition to protect against influenza virus in humans and domestic animals as well as in wild birds.

## Materials and Methods

### Materials

The 8-unit plasmid pYA4519 carrying all genes of influenza A virus (A/WSN/33) was described previously [51]. *S*. Typhimurium UK-1 strains χ3761, χ8276, χ8901, χ9052 and χ9909 were from the χ collection in the Curtiss laboratory. Plasmids pYA4784 and pYA3600 were from Curtiss lab plasmid stock. The *E*. *coli recA* mutant strain was purchased from Epicentre Biotechnologies (Madison, WI). All plasmids and bacterial strains used in this study are listed in Table 1. The primers used for plasmid construction are listed in Table 2. The specific pathogen-free (SPF) chicken embryonated eggs were purchased from Charles River Laboratories (Wilmington, MA). Madin-Darby canine kidney (MDCK) cells were purchased from ATCC.

**Table 1:** Plasmids and bacterial strains used in the study.

**Plasmid/strain**	**Description/genotype** *	**Source or reference**
Plasmids		
pBICEP-CMV3	pBR *ori*, *bla*, SV40 DTS	Sigma-Aldrich
p15A-PB2-kan	p15A *ori*, *cat*, *kan*, influenza PB2	[46]
pYA3342	pBR *ori*, *asd*	[54]
pYA4336	CMV-driven EGFP gene	This work
pYA4731	CMV-driven mCherry gene	[46]
pYA4545	pUC *ori*, *asd*, NF-κB binding site	[37]
pYA4784	p15A *ori*, *aroA*	Lab stock
pYA4519	p15A *ori*, *cat*, 8-unit	[46]
pYA4537	p15A *ori*, *cat*, 8-unit, SV40 DTS	This work
pYA4562	p15A *ori*, *cat*, 8-unit, SV40 DTS, NF-κB binding site	This work
pYA4913	p15A *ori*, *cat*, 7-unit (ΔHA)	This work
pYA4732	p15A *ori*, *cat*, 8-unit, CMV-driven mCherry gene	[46]
pYA4920	pBR *ori*, *asd*, 8-unit, SV40 DTS, NF-κB binding site	This work
pYA4954	p15A *ori*, *aroA*, 8-unit, SV40 DTS, NF-κB binding site	This work
pYA3600	Δ*aroA* suicide vector	Lab stock
*Salmonella* strains		
χ3761	wild type	[88]
χ9909	Δ*aroA*	χ collection
χ8276	Δ*asd*	[38]
χ8901	Δ*alr* Δ*dadB*	[89]
χ9052	Δ*alr* Δ*dadB* Δ*asd*	[89]
χ9834	Δ*alr* Δ*dadB* Δ*asd* Δ*recA*	This work
χ11018	Δ*alr* Δ*dadB* Δ*asd* Δ*recF*	This work
χ11150	Δ*alr* Δ*dadB* Δ*asd* Δ*recF* Δ*aroA*	This work
*E*. *coli* strain		
EPI300	F^−^ *mcrA* Δ*(mrr-hsdRMS-mcrBC)* Φ80d*lacZ* Δ*M15* Δ*lacX74 recA1 endA1 araD139* Δ*(ara*, *leu)7697 galU galK* λ^−^ *rpsL nupG trfA dhfr*	Epicentre Biotechnologies

* *bla*: ampicillin resistance gene;

*cat*: chloramphenicol resistance gene; *kan*: kanamycin resistance gene. Δ*aroA*: PABA and DHBA dependent; Δ*asd*: DAP dependent; Δ*alr* Δ*dadB* (also called *dadX*): D-alanine dependent; Δ*recA*: reduce plasmid recombination; Δ*recF*: reduce plasmid recombination.

**Table 2:** Primers used in this study.

**Primer**	**Sequence (5′→3′)** *	**Enzyme site**
P1	tgcgatcgctgtggaatgtgtgtcagttaggg	*Asi*SI
P2	accttgatcaggagctttttgcaaaagcctagg	*Eco*NI
P3	ccggaattgccagctggggc	
P4	actgacacacattccacagcgatcgctccgcgcacatttccccg	*Asi*SI
P5	cggggaaatgtgcgcggagcgatcgctgtggaatgtgtgtcagt	*Asi*SI
P6	accttgatcaggagctttttgcaaaagcctaggc	*Eco*NI
P7	cccgtaattgattactattgcggagttaggggcgggac	
P8	gtcccgcccctaactccgcaatagtaatcaattacggg	
P9	accttgatcaggcggaactccatatatggg	*Eco*NI
P10	taagccggctccaacatcacaggtaaacag	*Ngo*MIV
P11	ttagcgatcgccggaagatccgcacatctct	*Asi*SI
P12	taacccggggcgctagcggagtgtatac	*Xma*I
P13	ttagcgatcgcggctaaacgcgttgtttaac	*Asi*SI
P14	taagccggcccggaattgccagctggggc	*Ngo*MIV
P15	ttacacgtgtccgcgcacatttccccgaaaag	*Pml*I

* The restriction enzyme sites in primers are underlined.

### Plasmid construction

The 8-unit plasmid pYA4519 (Table 1) was first linearized with *Srf*I and treated with calf intestinal alkaline phosphatase (CIAP). Simian virus 40 (SV40) DNA nuclear targeting sequence (DTS) was amplified from plasmid pBICEP-CMV3 (Sigma-Aldrich) with primers P1 and P2 (Table 2). The amplified DNA fragment was treated with T4 polynucleotide kinase (T4 PNK) and ligated into *Srf*I/CIAP pretreated pYA4519, resulting in plasmid pYA4537. To construct plasmid pYA4562, the kanamycin resistance gene (*kan)* together with its promoter was amplified from plasmid p15A-PB2-kan with primers P3 and P4. SV40 DTS was amplified from pBICEP-CMV3 with primers P5 and P6. Both amplicons were fused by a third PCR using primers P3 and P6. The *kan*-DTS fusion was further amplified with primers P3 and P7. A synthetic NF-κB binding site in plasmid pYA4545 was amplified with primers P8 and P9. The two fragments were fused by PCR with primers P3 and P9 to obtain a fusion product *kan*-DTS-NFκB, which was treated with T4 PNK and inserted into *Srf*I/CIAP pretreated pYA4519 to obtain pYA4562.

To eliminate the need for any antibiotic resistance markers, a DNA fragment containing the *asd* gene and pBR *ori* was amplified from plasmid pYA3342 (Table 1) with primers P10 and P11 (Table 2). The PCR product was digested with *Ngo*MIV and *Asi*SI, and ligated into pYA4562 treated by the same enzymes to replace the region containing the p15A *ori*, *kan* and chloramphenicol resistance gene (*cat*). The resulting plasmid was designated pYA4920. Similarly, a DNA fragment containing the *aroA* gene and p15A *ori* was amplified from plasmid pYA4784 with primers P12 and P13, digested with *Xma*I and *Asi*SI, then inserted between the *Ngo*MIV (produces compatible end with *Xma*I-digested DNA) and *Asi*SI sites in pYA4562 to replace the p15A *ori*, *kan* and *cat*. The resulting plasmid was called pYA4954. To construct plasmid pYA4913, the *kan* cassette was amplified from plasmid p15A-PB2-kan with primers P14 and P15. The amplified *kan* cassette was trimmed by *Ngo*MIV and *Pml*I, and ligated into pYA4519 digested by same enzymes to replace the HA cassette. The resulting 7-unit plasmid pYA4913 (ΔHA) was used as a control. During plasmid construction, large DNA fragments were separated on agarose gels containing 10 μg/ml crystal violet and excised under bright room light [52]. The *E*. *coli recA* mutant strain was used for all DNA cloning experiments. Plasmid DNA was prepared using Mini and Maxi plasmid preparation kits (Qiagen).

### Strain construction and growth conditions

Each strain was cultured in LB broth with complementary supplements, such as 50 μg/ml diaminopimelic acid (DAP,) or 100 μg/ml DL-alanine. Strain χ9834 was generated from χ9052 by deleting the *recA* gene using Lambda Red recombinase-mediated recombination [53]. Strain χ11018, another χ9052-derivative, was created by deleting the *recF* gene using P22HT*int*-mediated transduction [54]. Suicide vector pYA3600 contains *aroA*-flanking regions from *S*. Typhimurium was designed to enable a 1296 bp in-frame-deletion of *aroA* ORF (*aroA*-12 to 1284) (Table 1). *S*. Typhimurium strain χ9909 was generated from the wild-type *S*. Typhimurium strain χ3761 using suicide vector pYA3600. Strain χ11018 was transduced with P22 lysates (χ9909::pYA3600) to delete the *aroA* gene [54]. The resulting strain was designated χ11150. Conditional growth of the strains was tested on LB agar plates supplemented with or without 50 μg/ml DAP and/or 100 μg/ml DL-alanine. It is worth noting that electroporation was employed to introduce plasmid and DNA fragment into *Salmonella* strains.

### Cell culture

Chicken embryonic fibroblasts (CEF) were prepared from 8-day-old SPF chicken embryos using 0.25% trypsin-EDTA. To passage the CEF, 0.05% trypsin-EDTA was used for digestion. CEF and MDCK cells were maintained in Dulbecco's modified Eagle's medium (DMEM, Invitrogen, Cat. 11885) supplemented with 10% fetal bovine serum (FBS), 100 U/ml penicillin and 100 μg/ml streptomycin. To co-culture CEF and MDCK cells, each type of nearly confluent cells grown in 75 cm^2^ flasks were first treated with trypsin. Then, 1/4 volume of MDCK cells were mixed with 1/6 volume of CEF. Growth medium was added to a total volume of 20 ml. The mixed cells were seeded into six-well plates (3 ml per well) and maintained at 37°C in 5% CO_2_. At all times, *Salmonella* strains were incubated with co-cultured cells in absence of penicillin and streptomycin.

### Virus generation via DNA transfection

To ascertain that the newly constructed plasmids are enable to recover influenza virus in avian cells, the co-culture of CEF and MDCK cells grown in 6-well plates were transfected using cationic lipids (Invitrogen, Cat. 11668019) according to the vendor’s instructions. Briefly, 2 μl of transfection reagent per μg plasmid DNA were individually diluted in 100 μl of minimal essential medium (MEM, Invitrogen, Cat. 31985088). After 5 min incubation at room temperature (RT), the diluted transfection reagent was mixed with the DNA, followed by 40 min incubation at RT. The transfection mixture was added to pre-washed eukaryotic cells drop by drop. After 3 h incubation, the transfection medium was replaced with 2 ml of MEM containing 0.3% bovine serum albumin (BSA), penicillin and streptomycin. At one day post transfection, each well was supplemented with 1 ml of MEM containing 2 μg/ml TPCK-trypsin, 0.3% BSA, penicillin and streptomycin. At three to five days post transfection, cell supernates were titrated onto MDCK cell monolayers to estimate influenza virus titers. The rescued influenza viruses were confirmed by hemagglutination assays [55], and by western blot with influenza A virus NP or M2-specific antibodies.

### Conditional destruction of *Salmonella* vectors

Each strain was grown in 5 ml of LB broth with complimentary supplement(s) at 30°C overnight. To determine *Salmonella* lysis in absence of necessary supplement(s), 1 ml of the overnight culture was pelleted and resuspended in 1 ml of LB broth. 200 μl of resuspended bacteria was transferred into 5 ml of LB broth. After overnight standstill incubation at 37°C, 1 ml of the culture was pelleted at 8,000 rpm for 5 min and resuspended in 50 μl of 1% NaCl. Live and dead bacteria were determined by differential staining using the bacterial viability kit (Life Technologies, Cat. L34856) followed by microscopy. Propidium iodide (PI) and green fluorescent nucleic acid stain were dissolved into 5 ml of 1% NaCl to prepare 2 × staining solution as described in the kit manual. Resuspended *Salmonella* cells were mixed with an equal volume of 2 × staining solution and incubated at RT for 15 min. Then, 5 μl of the stained bacteria were mixed with 5 μl of 37°C pre-warmed 1.0% low melting agarose (Sigma-Aldrich, Cat. A5030) in 1% NaCl. Immediately, 5 μl of mixture was transferred onto the center of a slide and covered with a coverslip. Gentle pressure was applied on the coverslip to form a thin layer of bacteria. The slide was set aside for 20 min at RT to solidify the agarose (to stop the moving bacteria and limit the spreading of released DNA). Bacteria were observed under a fluorescent microscope using dsRed and GFP filters. The ratios of PI-stained cells were calculated by counting more than 200 *Salmonella* cells for each strain. Chi-square tests were performed for statistical analysis.

### *Salmonella*-mediated plasmid delivery

*S*. Typhimurium strains carrying plasmid (pYA4336, pYA4731, pYA4732) were individually cultured in 3 ml of LB broth containing 50 μg/ml DAP and/or 100 μg/ml DL-alanine, along with 100 μg/ml ampicillin or 25 μg/ml chloramphenicol to maintain the plasmid. After overnight incubation at 30°C with vigorous shaking (200 rpm), 1 ml of the culture was harvested by centrifugation at 12,000 rpm for 2 min and the pellet was resuspended in 1 ml of DMEM devoid of any supplements. Plasmid pYA4336 (15 μg) was mixed with χ9052 to serve as control. CEF were washed three times with DMEM. Then, each well of cells (6-well plate) received 900 μl or 800 μl of DMEM, followed by 100 μl or 200 μl of resuspended bacteria to yield a total volume of 1 ml. After 1 h incubation at 37°C in a 5% CO_2_ humidified incubator, the infection medium was replaced with 3 ml of DMEM containing 10 μg/ml gentamicin and 10% FBS. At one day post infection, cells were fixed with 2% paraformaldehyde (PFA) and stained with 3 μM 4ʹ,6-diamidino-2-phenylindole dihydrochloride (DAPI). Cells synthesizing enhanced green fluorescent protein (EGFP) or red fluorescent protein (mCherry) were observed under a fluorescence microscope, and were counted in random fields [56].

### DNA recombination in *Salmonella*

*Salmonella* strains χ9052, χ9834 and χ11018 were each electroporated with plasmid pYA4519, plated onto LB agar plates and incubated overnight at 37°C. Single colonies from each electroporation were individually cultured in 3 ml of LB broth and incubated at 37°C overnight with rotary shaking. Plasmid DNA was prepared, and verified by *Bam*HI restriction mapping. The percentage of clones having the expected restriction pattern was calculated. Statistically significant differences were revealed by Chi-square tests. From each strain, a correct clone was diluted 1:1000 into 3 ml of LB broth and grown at 37°C for 12 h. The dilution and growth process was repeated for 4 additional cycles. Plasmid DNA was extracted from 1.5 ml of culture from each cycle of growth. An aliquot of plasmid from each sample was digested with *Bam*HI and separated on a 1.2% agarose gel. All media were supplemented with 25 μg/ml chloramphenicol to maintain the plasmid, 50 μg/ml DAP and 100 μg/ml DL-alanine to satisfy the growth requirements due to the Δ*asd*, Δ*alr* and Δ*dadB* mutations.

### Virus generation via *Salmonella*-mediated plasmid delivery

The required strains of *S*. Typhimurium were each inoculated into 3 ml of LB broth containing appropriate supplements. For χ9834 carrying pYA4519 or pYA4562, the medium was supplemented with 50 μg/ml DAP, 100 μg/ml DL-alanine and 34 μg/ml chloramphenicol. For χ11150, the medium was supplemented with 50 μg/ml DAP, 100 μg/ml DL-alanine, 10 μg/ml 2,3-dihydroxybenzoic acid (DHBA), and 10 μg/ml para-aminobenzoic acid (PABA). Strain χ11150(pYA4920) was incubated in similar medium, but without DAP. For χ11150(pYA4954), LB broth was supplemented with 50 μg/ml DAP and 100 μg/ml DL-alanine. After overnight incubation at 30°C with rotary shaking (200 rpm), 1 ml of the bacterial culture was pelleted at 12,000 rpm for 2 min and resuspended into 1 ml of DMEM without any supplement. 10 μg of plasmid pYA4920 was mixed with χ11150 to serve as control. The infection process was performed as described above. At one day post infection, 1 ml of MEM containing 0.3% BSA, 10 μg/ml gentamicin and 2 μg/ml TPCK-trypsin was added to each well. At five days post infection, the supernate was assayed for hemagglutination of 0.5% chicken red blood cells (RBC) and titrated on MDCK cells as 50% tissue culture infective dose (TCID_50_). The amplified virus was further verified by western blot with NP or M2-specific antibodies.

### Ethics Statement

The use of chicken embryonic fibroblasts did not require ethical approval.

## Results

### Construction of plasmid able to generate influenza virus in avian cells

**Fig 2:**
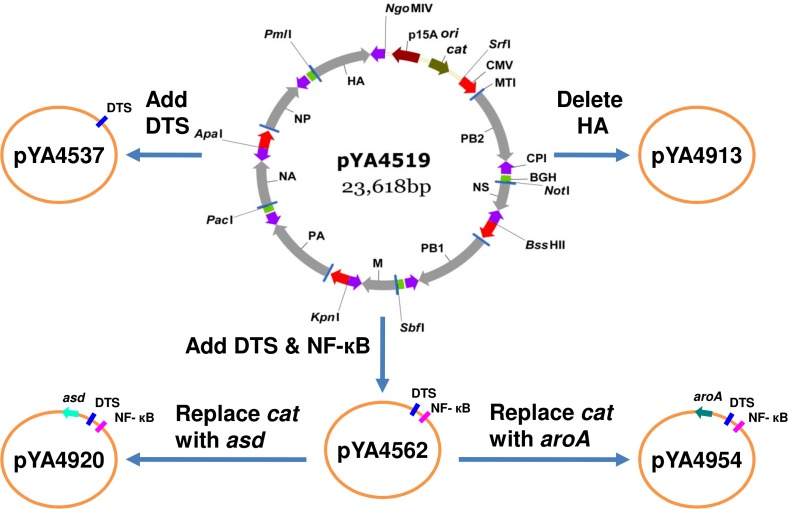
Construction of Asd^+^ and AroA^+^ vectors for generating influenza virus. Plasmid pYA4519 carries eight influenza WSN virus genes, which are individually flanked by chicken RNA polymerase I promoter (CPI) and murine RNA polymerase I terminator (MTI) to generate vRNAs. The viral polymerase subunits (PB1, PB2 and PA) and nucleoprotein (NP) genes are further flanked with CMV promoter and bovine growth hormone gene polyadenylation sequence (BGH) to generate mRNAs, and eventually synthesize proteins. The SV40 DTS was inserted into pYA4519 at the *Srf*I site to generate pYA4537. A DNA fusion of SV40 DTS, NF-κB binding site and *kan* cassette was inserted into pYA4519 at the *Srf*I site, resulting in plasmid pYA4562. The DNA fragment containing the *asd* gene and pBR *ori* was ligated into pYA4562 to replace the DNA fragment containing the p15A *ori*, *cat* and *kan*, resulting in plasmid pYA4920. Plasmid pYA4954 was derived from pYA4562 by replacing the *cat*-*kan* fragment with the *aroA* cassette. The plasmid-borne *asd* and *aroA* genes complement Δ*asd* and Δ*aroA* mutations in *Salmonella* hosts, therefore, eliminating the use of antibiotic resistance genes. The HA cassette was deleted from pYA4519 to generate a 7-unit plasmid pYA4913, which was used as a control. For simplicity, only plasmid pYA4519 was shown in detailed map.

The 8-unit plasmid pYA4519 encodes the complete set of vRNAs, polymerase subunits (PB1, PB2 and PA) and nucleoprotein (NP) of influenza WSN virus [46]. Upon transfection, this 8-unit plasmid generates influenza virus in CEF. To achieve more efficient virus generation, we introduced the SV40 DTS (144 bp) into plasmid pYA4519, resulting in plasmid pYA4537 (DTS, 8-unit) as illustrated in Fig. 2. The binding site for NF-κB and SV40 DTS were both introduced into pYA4519 to generate plasmid pYA4562 (DTS, NF-κB, 8-unit). To eliminate the use of antibiotics, *asd* and *aroA* genes were used as selective markers and were individually introduced into pYA4562 to obtain plasmids pYA4920 (*asd*, DTS, NF-κB, 8-unit) and pYA4954 (*aroA*, DTS, NF-κB, 8-unit), respectively. The two plasmids will complement the Δ*asd* or Δ*aroA* mutation in the *Salmonella* carrier. The HA cassette was deleted from pYA4519 to obtain plasmid pYA4913 (ΔHA, 7-unit), which serves as the negative control in virus reconstitution experiments. All resulting plasmids are listed in Table 1. To confirm that the newly constructed 8-unit plasmids are functional as designed, each of these plasmids was introduced into a co-culture of CEF and MDCK cells. Of note, the MDCK cells were employed to propagate influenza virus generated in and released from CEF [46]. On day 3 and day 5, viruses were generated at the titers of 10^3^–10^7^ TCID_50_/ml (Table 3). The viruses thus generated were confirmed to be influenza virus by hemagglutination assay and by immunoblotting with influenza NP or M2-specific antibodies (data not shown).

**Table 3:** Influenza virus generation from transfected MDCK-CEF co-cultures.

**Plasmid**	**Titer (TCID_50_/ml)**	
	**3^rd^ day**	**5^th^ day**
pYA4519	1.0 × 10^7^	3.2 × 10^7^
pYA4537	1.0 × 10^6^	1.0 × 10^8^
pYA4562	3.2 × 10^5^	3.2 × 10^8^
pYA4920	1.0 × 10^4^	3.2 × 10^7^
pYA4954	3.2 × 10^5^	3.2 × 10^6^
pYA4913	0	0

### *Salmonella*-mediated DNA delivery into cultured avian cells

**Fig 3:**
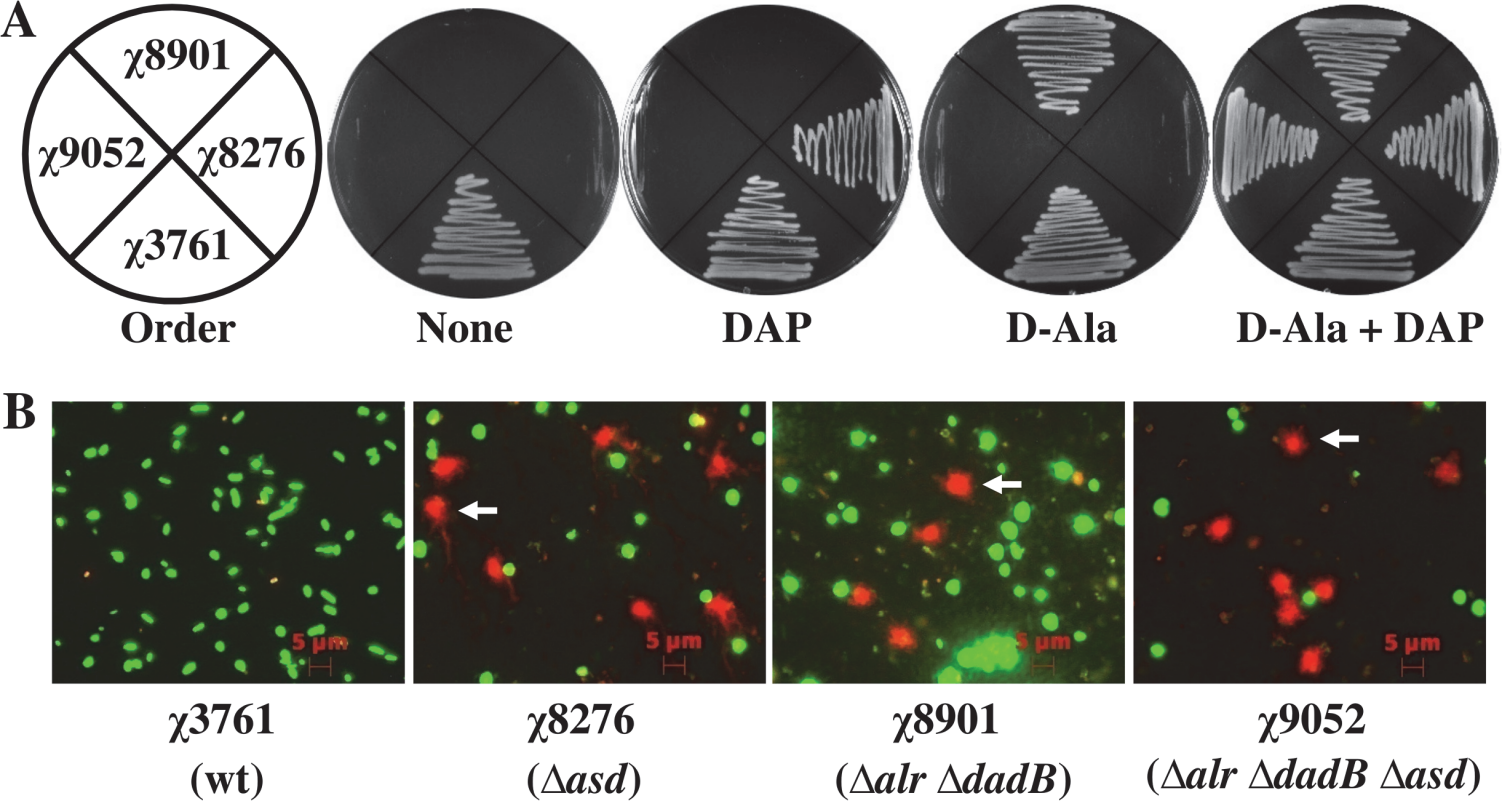
Conditional survival and DNA release from *Salmonella* strains. (A) *Salmonella* strains were streaked on LB-agar plates with or without 50 μg/ml DAP and/or 100 μg/ml DL-alanine (D-Ala). *Salmonella* growth was observed after overnight incubation at 37°C. The streak order is given to the left. (B) Overnight culture of *Salmonella* was collected and resuspended in LB medium without any supplements. After overnight static growth at 37°C, cells were stained with PI and green fluorescent nucleic acid stain. Live cells are stained green, and the dead cells are stained red. Strain number and the genotype are given under each panel. Enlarged cell size and release of cell DNA content (indicated by arrow heads) were observed in the case of auxotrophic strains, but not in the wild-type control strain χ3761.

*Salmonella* strains χ8276 (Δ*asd*) and χ8901 (Δ*alr* Δ*dadB*) only grew on LB agar supplemented with DAP or DL-alanine, while the strain χ9052 (Δ*asd* Δ*alr* Δ*dadB*) relied on both supplements (Fig. 3A). To verify the conditional destruction of *Salmonella* strains, each strain was incubated overnight in LB broth lacking DAP and DL-alanine supplements. Green fluorescent nucleic acid stain and PI were used to stain bacterial cells. Live cells can only be stained with green fluorescent nucleic acid stain and exhibit green fluorescence (Fig. 3B). The impaired bacterial membrane becomes permissible to PI, therefore, these cells are stained with green fluorescent nucleic acid stain and PI, showing red fluorescence due to dominant PI staining. Few cells stained yellow due to their slightly impaired membrane that allowed minute quantities of PI to enter the cell. The yellow fluorescence was resulted from the combination of red and green fluorescence at an appropriate ratio. Significantly higher ratios (*P* < 0.001) of red cells were observed in the Δ*asd* mutant (χ8276, 22.2%), the Δ*alr* Δ*dadB* mutant (χ8901, 8.2%) and the Δ*asd* Δ*alr* Δ*dadB* mutant (χ9052, 25.5%) than in wild-type strain χ3761 (2.2%), indicating membrane permeability and lysis by virtue of the various mutations introduced into their genomes. The DAP-dependent strains χ9052 (Δ*asd* Δ*alr* Δ*dadB*) and χ8276 (Δ*asd*) showed similar percentages of red cells (*P* > 0.05), but significantly higher than the percentage of red cells shown by the D-alanine-dependent strain χ8901 (Δ*alr* Δ*dadB*) (*P* < 0.001). Using this protocol, some membrane-impaired cells were also stained while they were releasing PI-bound DNA (arrow). Empty bacterial envelopes, which have lost their DNA content, were observed in strains χ8276 (Δ*asd*), χ8901 (Δ*alr* Δ*dadB*) and χ9052 (Δ*asd* Δ*alr* Δ*dadB*) under bright field microscopy but were not visible with the fluorescent filters (data not shown).

**Fig 4:**
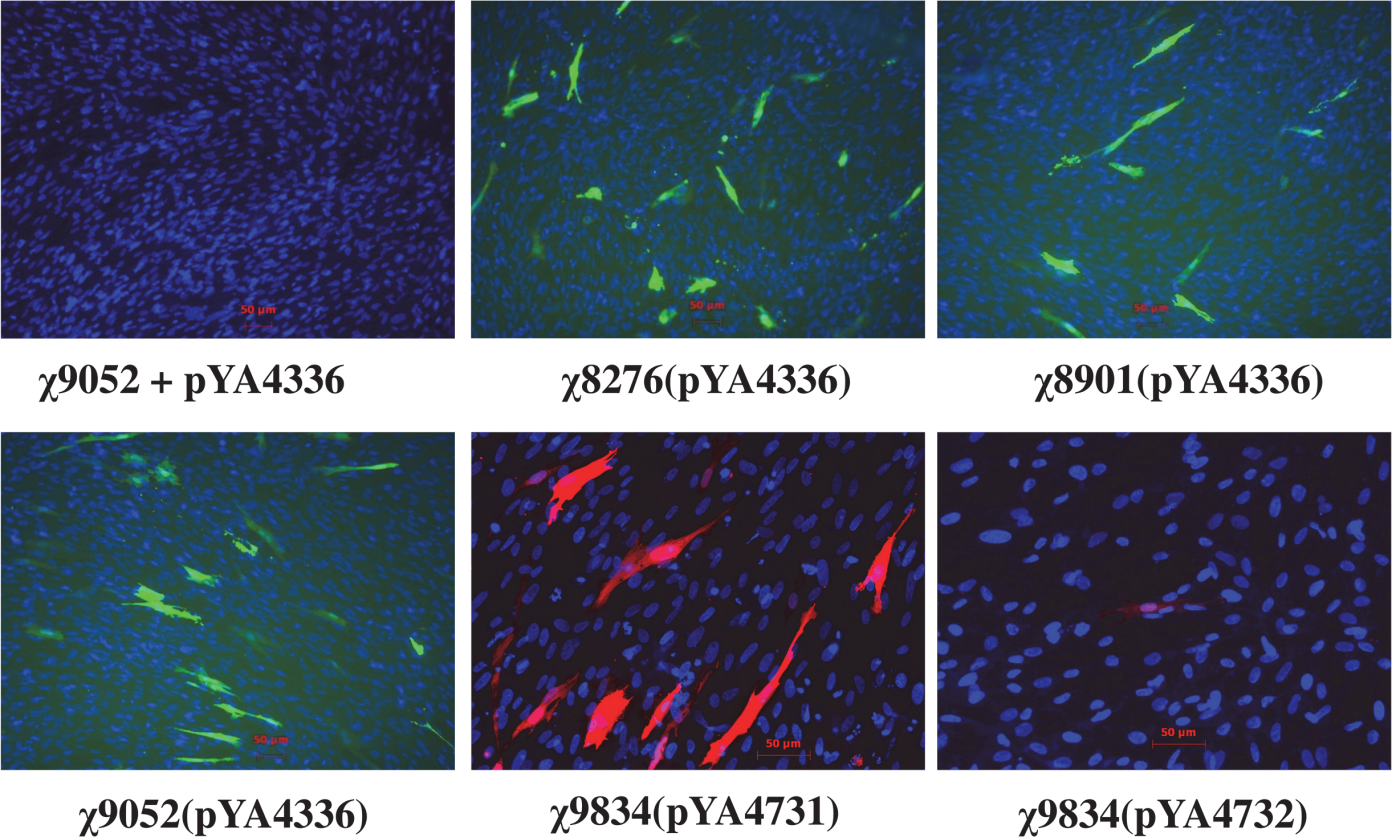
*Salmonella*-mediated plasmid delivery into CEF. CEF were infected with *Salmonella* strains each carrying a plasmid encoding EGFP or mCherry under regulation of a CMV promoter. As a control, CEF were incubated with a mixture of χ9052 and pYA4336 DNA. At 24 h post infection, the cells were fixed and then stained with DAPI to show the nucleus (blue). Fluorescence by EGFP (green) and mCherry (red) were recorded under a fluorescence microscope. Strain number and the harbored plasmid are given under each panel. χ8276: Δ*asd*; χ8901: Δ*alr* Δ*dadB*; χ9052: Δ*alr*Δ*dadB* Δ*asd*; χ9834: Δ*alr* Δ*dadB* Δ*asd* Δ*recA*.

The intracellular delivery of plasmids was examined using different *Salmonella* strains. Here we used plasmids encoding EGFP or mCherry under control of a cytomegalovirus (CMV) promoter, including 6 kb plasmid pYA4336 (EGFP), 6 kb plasmid pYA4731 (mCherry), and a 25 kb plasmid pYA4732 (mCherry, 8-unit) (Table 1). Synthesis of EGFP/mCherry in avian cells indicates the successful plasmid delivery into target cells by the *Salmonella* carrier. For some reason, we consistently observed that culturing *Salmonella* at low temperature (RT or 30°C) instead of 37°C is critical for successful plasmid delivery into cultured CEF (data not shown). Using the protocol described in the Methods section, plasmid pYA4336 (EGFP, 6 kb) was delivered into CEF by each of the strains χ8276 (Δ*asd*), χ8901 (Δ*alr* Δ*dadB*) and χ9052 (Δ*asd* Δ*alr* Δ*dadB*) as indicated by CEF synthesizing EGFP (Fig. 4). No EGFP synthesis in CEF was observed when incubated with strain χ9052 (Δ*asd* Δ*alr* Δ*dadB*) mixed with plasmid pYA4336 (EGFP, 6 kb), suggesting that plasmid carriage by *S*. Typhimurium strains was required for delivery of the plasmid into host cells. We then tested the ability of χ9834 (Δ*asd* Δ*alr* Δ*dadB* Δ*recA*) to deliver large plasmids into CEF. Two different wells of CEF were individually incubated with χ9834 carrying either 6 kb plasmid pYA4731 or 25 kb plasmid pYA4732 each expressing the mCherry gene (Fig. 4, lower panel). The χ9834(pYA4731) resulted in 4.4% of mCherry-positive CEF. Consistently, less than 10 CEF in a 10 cm^2^ well were found to synthesize mCherry after χ9834(pYA4732) infection, suggesting that the larger plasmid has limited successful delivery into the avian cells.

### Improving DNA stability in *Salmonella* carrier by including a *rec* mutation

**Fig 5:**
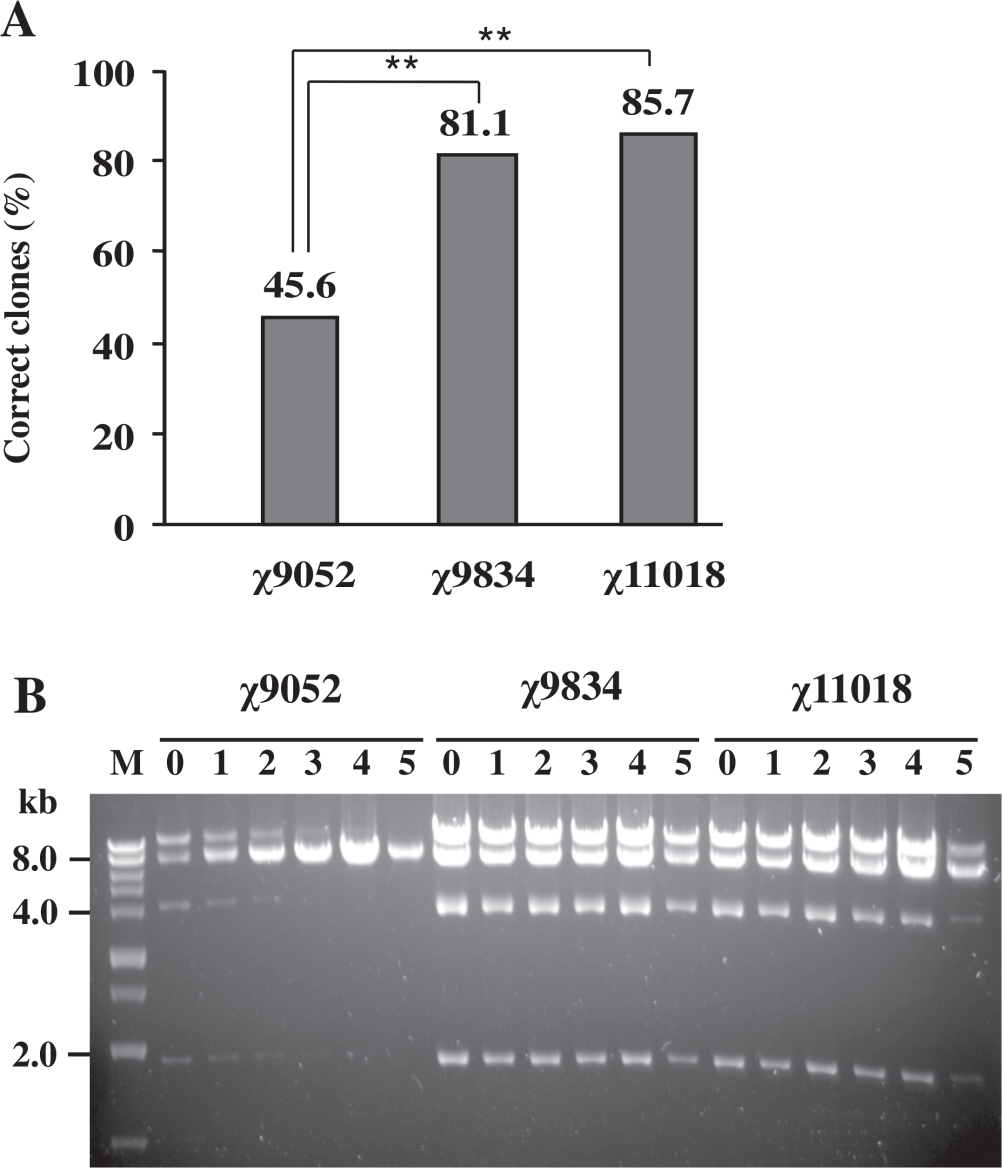
Intraplasmid recombination of pYA4519. (A) *Salmonella* strains χ9052 (Δ*alr* Δ*dadB* Δ*asd*), χ9834 (Δ*alr* Δ*dadB* Δ*asd* Δ*recA*) and χ11018 (Δ*alr* Δ*dadB* Δ*asd* Δ*recF*) were electroporated with plasmid pYA4519. Plasmid DNA from electroporants was evaluated by *Bam*HI digestion, resulting in four DNA fragments of 10354 bp, 7285 bp, 4013 bp, and 1966 bp from a correct plasmid. For strains χ9834 and χ11018, the percentages of correct clones are over 80%, which are significantly higher than that for parental strain χ9052 (*P* < 0.01, **). (B) *Bam*HI digests of pYA4519 DNA isolated from passaged electroporants of strains χ9052, χ9834 and χ11018. A correct clone was chosen according to the *Bam*HI restriction map (lane 0). For each passage, bacteria were diluted 1:1000 and cultured at 37°C for 12 h. This process was repeated for a total of five cycles. The plasmids were extracted and digested by *Bam*HI (lanes 1–5).

One feature of the influenza virus-generating plasmids is the presence of repetitive homologous sequences in the promoter and the terminator elements flanking each of the viral genes (Fig. 2). These homologous sequences form ideal substrates for DNA recombination, which might negatively impact plasmid stability. To stabilize these plasmids in an attenuated *Salmonella* vector, the recombinase (RecA or RecF) gene was deleted from the Rec^+^ strain χ9052 (Δ*asd* Δ*alr* Δ*dadB*), resulting in strains χ9834 (Δ*asd* Δ*alr* Δ*dadB* Δ*recA*) and χ11018 (Δ*asd* Δ*alr* Δ*dadB* Δ*recF*). Plasmid pYA4519 was electroporated into each of the three strains. Recombination was monitored by restriction enzyme digestion of plasmid DNA isolated from different strains. After the initial electroporation, we analyzed *Bam*HI-digested DNA from > 50 individual colonies from each strain. Our results (Fig. 5A) indicated that less than half of the clones in the Rec^+^ strain contained plasmids with the expected restriction pattern, while over 80% of the plasmids obtained from either the Δ*recA* or Δ*recF* strain remained unaltered (*P* < 0.01 compared to the Rec^+^ strain).

A single correct colony from each electroporated strain was grown in LB broth and passaged five times at 1000-fold dilutions. After each passage, plasmid DNA was extracted from the culture and analyzed by *Bam*HI digestion, followed by agarose gel electrophoresis (Fig. 5B). We noted that at time 0, before passage, the plasmid yield from the Rec^+^ strain (χ9052) was less than that obtained from the two *rec* mutants. After the second cycle of growth, there was a reduction in the amount of DNA in three of the expected bands, indicating that the plasmid structure was perhaps deteriorating at each passage. Qualitative evaluation of the DNA bands on agarose gels suggested that the plasmid structure was stable for the first four passages in strains χ9834 (Δ*recA*) and χ11018 (Δ*recF*). This assay was repeated three times, and we obtained consistent results each time.

### Virus generation via *Salmonella*-mediated plasmid delivery

*S*. Typhimurium strain χ9834 (Δ*alr* Δ*dadB* Δ*asd* Δ*recA*) carrying plasmid pYA4519 or pYA4562 was first employed to infect co-cultures of CEF and MDCK cells. As shown in Table 4, in five out of six wells of infected cells, the presence of influenza virus was verified by titration on MDCK cell monolayers as well as by hemagglutination assay using chicken red blood cells. It seemed that plasmid pYA4562 carrying the DTS and NF-κB binding site resulted in higher titers of influenza virus than its parental plasmid pYA4519. Based on this observation, plasmid pYA4562 was used to construct plasmids pYA4920 and pYA4954, which are free of antibiotic resistance genes.

**Table 4:** Influenza virus generation by *Salmonella*-mediated plasmid delivery.

**Group**	**Dose^$^**	**Experiments (No.)**									
		**1**		**2**		**3**		**4**		**5**	
		**H**	Titer*	**H**	**Titer**	**H**	**Titer**	**H**	**Titer**	**H**	**Titer**
χ9834(pYA4519)	100 μl	0	0	-	-	-	-	-	-	-	-
	200 μl	0	10^3^	-	-	-	-	-	-	-	-
	500 μl	1:2	10^5^	-	-	-	-	-	-	-	-
χ9834(pYA4562)	100 μl	1:16	10^6^	-	-	-	-	-	-	-	-
	200 μl	1:4	10^5^	-	-	-	-	-	-	-	-
	500 μl	1:8	10^6^	-	-	-	-	-	-	-	-
χ11150(pYA4920)	100 μl	-	0	0	0	0	0	1:128	10^7^	0	0
	200 μl	-	0	0	0	1:32	10^7^	0	0	1:32	10^7^
χ11150(pYA4954)	100 μl	-	10^7^	1:64	10^7^	1:16	10^7^	0	0	0	0
	200 μl	-	0	0	0	0	0	0	0	0	0
χ11150+pYA4920	100 μl	-	0	0	0	0	0	0	0	0	0
	200 μl	-	0	0	0	0	0	0	0	0	0

^$^: represent different amount of *Salmonella* used for cell infection.

H: Hemagglutination assay of supernates from *Salmonella*-infected CEF/MDCK cell co-culture.

*: Infectious virus titer measured as TCID_50_/ml.

-: Not performed.

*S*. Typhimurium strain χ11150 (Δ*alr* Δ*dadB* Δ*asd* Δ*recF* Δ*aroA*) harbors either pYA4920 (*asd*, DTS, NF-κB, 8-unit) or pYA4954 (*aroA*, DTS, NF-κB, 8-unit) to complement the Δ*aroA* or Δ*asd* mutation, respectively. Strain χ11150(pYA4920) was grown in LB medium supplemented with DL-alanine, DHBA and PABA, whereas strain χ11150(pYA4954) was grown in LB medium supplemented with DAP and DL-alanine. Strain χ11150 (not carrying any plasmid) was grown in LB medium supplemented with DAP, DL-alanine, DHBA and PABA.

Strains χ11150(pYA4920) and χ11150(pYA4954) were each incubated with co-cultures of CEF and MDCK cells. The virus generation was verified by titration on MDCK cells and hemagglutination assays. For CEF/MDCK co-cultures infected with χ11150(pYA4920) and χ11150(pYA4954), 6 out of the 20 cell supernates were detected with influenza virus with estimated titers of 10^7^ TCID_50_/ml (Table 4). The hemagglutination titers ranged from 1:16 to 1:64. A mixture of plasmid DNA with the *Salmonella* carrier (χ11150 + pYA4920) did not result in any hemagglutination-positive or cytopathic effect (CPE)-causing cell supernate, once again confirming that the plasmid delivery into the eukaryotic host is *Salmonella*-dependent. The generated infectious particles were confirmed to be influenza A virus by western blot with NP or M2-specific antibodies (data not shown).

## Discussion

Influenza, a zoonotic disease, is one of the most serious threats to human, poultry and wild birds worldwide. Administration of a vaccine orally or by spray could be instrumental in mass immunization of poultry and maybe wild birds to substantially reduce the reservoir of virus in birds, thus eventually lessening bird-to-human transmission of avian influenza virus and preventing an influenza epidemic/pandemic. Currently available veterinary influenza vaccines are not cost-effective for such a large-scale production and involve labor-intensive processes. Our goal is to develop a bacterial-based vaccine technology that can overcome each of these limitations. Briefly, an attenuated bacterial carrier delivers a target plasmid that encodes the entire set of vRNA and proteins for assembling attenuated influenza viruses or pseudovirus particles in the host cells. The virus generated *in vivo* thus serves as the vaccine to induce protective immunity. To examine the feasibility of developing such a technology and to identify potential limiting factors in its application, we chose *S*. Typhimurium as the delivery vector and influenza WSN strain as the model virus. We had previously developed a single 8-unit plasmid that encodes WSN viral components for virus generation [46]. In this study we delivered the plasmid and its derivatives into CEF using genetically modified attenuated strains of *S*. Typhimurium and quantified the virus generated in CEF, providing initial proof-of-concept to the hypothesized vaccine. The study also identified critical factors that limit the development of influenza vaccine based on *Salmonella*-mediated plasmid delivery.

*Salmonella*, a genus of *enterobacteria*, is a free-living bacterium capable of host cell invasion [57–59], a property that was widely explored for plasmid delivery into target host cells [60]. More efficient plasmid delivery can be obtained with auxotrophic strains [61,62]. In this study we used *S*. Typhimurium strains carrying loss-of-function mutation(s) in genes encoding enzymes critical to maintain cell wall structure or in metabolism (Table 1). The *asd* gene encodes aspartate-β-semialdehyde dehydrogenase, an enzyme required for bacterial cell wall synthesis. Inactivation of the *asd* gene causes an obligate requirement for DAP [63,64]. The *alr* and *dadB* (also called *dadX*) genes encode alanine racemases, which are responsible for the racemization of another key cell wall component, D-alanine [65]. Neither DAP or D-alanine are present in LB broth, in the routinely used cell culture media or in the animal tissues, and thus require external supplementation. Upon deprivation of DAP or D-alanine, *Salmonella* strains containing corresponding mutation(s) undergo cell wall-impaired lysis (Fig. 3). AroA is one key enzyme in the pathway to synthesize L-tryptophan, tyrosine, phenylalanine and *p*-aminobenzoate [66]. Hence survival of the *Salmonella* strain containing Δ*aroA* mutation requires amino acid supplements in the growth medium.

In this study, two combinations of auxotrophic mechanisms were employed to facilitate *Salmonella*-mediated delivery of the 8-unit influenza plasmids. As to χ11150-mediated delivery of pYA4920, Δ*aroA*, Δ*alr* and Δ*dadB* conferred auxotrophic lysis. Δ*asd* in χ11150 was complemented by the wild-type *asd* gene on plasmid pYA4920, ensuring the stable maintenance of the plasmid in the *Salmonella* vector without the use of any antibiotics [64]. Regarding χ11150-mediated delivery of pYA4954, Δ*asd*, Δ*alr* and Δ*dadB* conferred cell wall-less lysis, and Δ*aroA* was complemented by wild-type *aroA* gene in the plasmid [67]. The same mechanism was also applied to χ9834-mediated delivery of pYA4519 and pYA4562, two plasmids carrying the chloramphenicol resistance gene (*cat*). Consistent with our previous findings [48], Δ*recA* and Δ*recF* mutations significantly stabilized the 8-unit plasmids in the *Salmonella* delivery vector (Fig. 5). As illustrated in Fig. 1, influenza virus was generated through *Salmonella*-mediated delivery of the plasmid into co-cultured avian cells (Table 4).

To serve as a vaccine it is critical that one dose of recombinant *Salmonella* produce enough quantity of attenuated influenza virus to induce protective immunity. Thanks to the immediate propagation of the newly reconstituted viruses by MDCK cells in the co-cultures, we detected influenza virus from 11 out of 26 CEF/MDCK co-cultures infected by recombinant *Salmonella* carrying the 8-unit influenza plasmid (Table 4). However, no detectable influenza virus was recovered in CEF-only cultures (not co-cultured with MDCK cells) infected by the recombinant *Salmonella* carrying the 8-unit plasmid (data not shown). The *in vitro* system could be optimized to obtain consistent virus generation from infected co-cultures, but the data presented here more realistically indicated that the current *Salmonella*-plasmid delivery system was not efficient in generating influenza virus.

Certain rate-limiting steps have to be overcome before implementing this strategy for vaccine development. (i) Nuclear import has been appreciated as one rate-limiting step during transfection, and was successfully overcome with small size plasmids by using nuclear localization signal peptide [68,69] or by introducing DNA nuclear targeting sequence (DTS) and NF-κB binding sites [70–73]. Use of SV40 DTS and NF-κB binding site slightly increased virus generation in *Salmonella*-mediated plasmid delivery (Table 4, χ9834(pYA4519) vs. χ9834(pYA4562)). However, this effect was not seen in virus generation during transfection experiments (Table 3, pYA4519 vs. pYA4562). (ii) The success of this strategy directly relies on the plasmid delivery efficiency of the *Salmonella* vector. Nevertheless, the efficiency of *Salmonella*-mediated delivery is low as demonstrated with the EGFP/mCherry-encoding plasmids (Fig. 4). This limitation might be overcome by including genetic modifications that enhance *Salmonella* invasion, intracellular lysis or plasmid release. Additionally, we found that only the *Salmonella* vector cultured at 30°C or lower temperature instead of 37°C exhibited successful delivery of reporter plasmids (EGFP or mCherry encoding plasmids). Understanding the mysterious mechanism may help design a bacterial vector with higher plasmid delivery capability. (iii) *Salmonella* delivery vector should be able to efficiently invade into host cells *in vivo*. The current attenuated strains of *S*. Typhimurium undergo direct lysis in tissue culture or *in vivo* upon depletion of necessary supplements. After chickens were intranasally or intramuscularly infected with *Salmonella* strains carrying the 8-unit plasmid, no influenza virus or influenza specific antibodies were detected (data not shown). We believe that instant clearance of inoculated *Salmonella* by the host is at least one of the governing factors.

SV40 and polio virus have been reconstituted as result of spontaneous transfer of viral genome/cDNA-containing plasmid from *E*. *coli* to cultured monkey cells [74,75]. In these studies, virus generation are very inefficient with merely resuspended bacteria, and could be improved by precipitating *E*. *coli* with calcium phosphate or by treating *E*. *coli* protoplasts with polyethylene glycol [74,75]. In another strategy, *E*. *coli* is engineered to express the invasin gene of *Y*. *pseudotuberculosis* and the listeriolysin O gene of *L*. monocytogenes [76]. The engineered *E*. *coli* can transfer its cargo, an artificial bacterial chromosome containing murine cytomegalovirus genome (MCMV BAC), into cultured murine fibroblasts, and results in generation of MCMV [77]. *In vivo* reconstitution of MCMV are achieved by injecting immunocompromised mice with *S*. Typhimurium carrying MCMV BAC [78]. We engineered *S*. Typhimurium to overexpress the invasin gene, aiming to arm the *Salmonella* vector with an additional invasion system. However, there was no apparent improvement towards *Salmonella*-mediated delivery of plasmid pYA4336 (Data not shown). *Salmonella* secreting listeriolysin protein or containing *sifA* mutation can disrupt the phagosomal membrane, and escape into the cytoplasm [79–81]. It is reasonable to question that whether these measures could promote *Salmonella*-mediated plasmid delivery.

Although bacterial vaccines have various advantages as mentioned above, biosafety concerns need to be addressed. Infrequently, genes can be transmitted among bacteria through transduction, transformation, or by conjugation. Transfer of antibiotic resistance genes may contribute to the undesirable spread of antibiotic resistance determinants [82]. Take this into consideration, the final vaccine will be based on the antibiotic resistance free systems, such as using Asd^+^ and AroA^+^ as markers in the plasmids. We also attenuated the *Salmonella* vector using multiple deletion mutations to prevent any phenotypic reversion. For example, the Δ*asd*, Δ*alr* and Δ*dadB* mutations contribute to attenuation of χ11150 (pYA4954) via multiple ways, so do the Δ*alr*, Δ*dadB* and Δ*aroA* mutations in χ11150(pYA4920). These strains are only viable in medium containing supplements that are unavailable in natural conditions.

We used the influenza WSN strain as a model virus in this study. For a vaccine based on this system, the plasmid will reconstitute an attenuated or replication-deficient influenza virus with HA and NA from a circulating influenza A virus [83–85]. Additionally, the polybasic cleavage peptides of the HA proteins are required for high pathogenicity of influenza viruses [4]. Thus, the polybasic cleavage site has to be eliminated during vaccine development [86,87], to prevent formation of a new reassortant virus with the other segments from a preexisting influenza virus in the host.

In conclusion, we prove that it is feasible to generate influenza virus through *Salmonella*-mediated plasmid delivery into cultured avian cells. Whereas, further improvements are necessary for the current *Salmonella* delivery system to achieve efficient virus recovery, a critical factor to induce protective immunity against influenza.
